# Sub-Aperture Synthetic Aperture Radar Imaging of Fixed-Platform Beam-Steering Radar for Blast Furnace Burden Surface Detection

**DOI:** 10.3390/s24144479

**Published:** 2024-07-11

**Authors:** Lifu Deng, Xianzhong Chen, Qingwen Hou

**Affiliations:** 1 School of Automation and Electrical Engineering, University of Science and Technology Beijing, Beijing 100083, China; b20200291@xs.ustb.edu.cn (L.D.); houqw@ustb.edu.cn (Q.H.); 2 Key Laboratory of Knowledge Automation for Industrial Processes of Ministry of Education, Beijing 100083, China

**Keywords:** blast furnace (BF), beam steering radar, burden surface imaging, non-linear spatial correction, trimmed geometric mean order likelihood CFAR (TGMOL-CFAR)

## Abstract

Due to the scheme of fixed-platform beam-steering radar and the space of the blast furnace being subjected to harsh environmental influences, the traditional detection methods of burden surface are limited by geometric distortion, noncoherent clutter, and noise interference, which leads to an increase in the image entropy value and the equivalent number of views, makes the density distribution of burden surface show a diffuse state, and greatly affects the stability and accuracy. In this paper, a new fixed-platform beam-steering radar synthetic aperture radar imaging method (FPBS-SAR) is proposed in the sensory domain of the blast furnace environment. From the perspective of fixed-platform beam-steering radar motion characteristics, the target range–azimuth coupled distance history model under the sub-aperture is established, the azimuthal Doppler variation characteristics of the fixed-platform beam-steering process are analyzed, and the compensation function of the transform domain for geometric disturbance correction is proposed. For noncoherent noise suppression in blast furnaces, the trimmed geometric mean-order-likelihood CFAR method is proposed to take into account the information of burden surface and clutter suppression. To verify the method, point target simulation and imaging for the industrial field measurement data are carried out. The results indicate that geometric distortion is well eliminated, the image entropy value and the equivalent number of views have decreased, and noncoherent noise in blast furnaces is suppressed.

## 1. Introduction

BF is a typical large black box countercurrent continuous reaction vessel; continuous blast, cycle charge, and the production process are accompanied by a complex physical and chemical reaction process. Its energy consumption accounts for about 60% of the total energy consumption of the iron and steel industry; CO_2_ emissions accounted for 90% [1,2]. The state of the cohesive zone and the updating of the dead pillaring of the furnace hearth reflect the activity of the oxidation-reduction reaction process in the generation of molten iron. Meanwhile, morphological distribution is of great significance to the adjustment of the charging strategy and the distribution of the gas flow, which is of great significance to the lowering of the energy consumption and the reduction in carbon emissions to ensure smooth operation and the enhancement of production [3,4].

Owing to the tuyere of continuous blasting, the top of the BF is in the environment of high temperature, high pressure, and high dust [5]. Because of the water-spraying process, dust combines with water vapor to form a high adherence to granular impurities, and the upper BF lighting conditions are fluctuations. Currently, methods for detecting the morphological distribution of the BF burden surface include machine vision imaging methods and radar imaging methods. Machine vision imaging methods install industrial endoscopes at the top of the BF to obtain the burden surface. Zhu et al. [6] proposed real-time burden surface video streams for industrial endoscope to estimate material surface contour and depth information, which is reconstructed for 1–2 m burden surface with better results, but for situations where there is a deep burden surface due to abnormal furnace conditions, the burden surface detection ability is poor. Huang et al. [7] proposed topography measurement and the completion method of 3D burden surface, improved accuracy in the region of the burden surface based on the texture energy feature and the relationship between the function and the actual depth. The method achieves endoscopic image enhancement through encoding and decoding the network structure, enhancing the information of the burden surface while greatly enhancing the central airflow in the image, reducing the need for shape estimation of the central area of the burden surface. Huang et al. [8] proposed a depth estimation method based on a single image to solve the depth estimation of the burden surface in the case of endoscopic image edge dispersion, which is effective in a calm and stable state, with good lighting conditions and strong airflow scenarios in the BF. Methods of machine vision are limited by the light conditions, dust concentration, conditions of the complex furnace, and interference conditions of the detection ability of burden surface to be improved. Chen et al. [9] proposed a 3-D imaging system with a six-point radar array, and by installing multiple radars in the BF roof windshield, the morphological features of the burden surface were obtained by a 3-D reconstruction method, but the resolution of reconstructing the 3-D burden surface morphology was low due to the limitation of the installation location and the number of radar installations. Tian et al. [10] constructed an FMCW mechanically steering radar detection system for BF burden surface, and the proposed B-mode imaging method to reconstruct the burden surface obtained better results, but the aperture synthesis and incoherent noise suppression ability for the radar steering process need to be improved. Zankl et al. [11] proposed a BF array radar system, and the effectiveness of the system was verified by simulation tests in cold, atmospheric pressure, ambient temperature, and dust-free environments, but no consideration was given to the measurement of the burden surface morphology and the suppression of incoherent interference under continuous operation of the BF. Online measurement of the morphology distribution of the burden surface during the production process needs to overcome three challenges, which can be summarized as follows.

- BF is a huge closed reaction vessel, and the installation position, size, number, and motion of the detection device are strictly limited.
- The light intensity inside the BF fluctuates greatly.
- High temperature, high pressure, high dust, and strong noise all have a significant impact on the imaging process.

Radar, as an active sensor with the ability to acquire high-resolution imaging in all-day, all-weather environments, has been widely used in the fields of terrain, climate, and environmental monitoring [12]. Synthetic aperture radar (SAR) is a two-dimensional imaging system widely used for high-resolution imaging of ground-based, airborne, and satellite-based target detection. Beam-steering SAR is a class of radar data acquisition process beam steering with the radar operating system; the satellite-based, airborne SAR scanning mode, sliding spotlight mode, and terrain observation by progressive scan (TOPS) mode are used to enhance the azimuthal resolution of the beam steering at the same time to enhance the wide-area imaging capability. In recent decades, the research work of SAR imaging has focused on different radar imaging mechanisms, imaging methods, coherent spotlight, clutter, and noise suppression methods. For satellite-carried SAR due to the satellite orbit and Earth orbit curved, the footprint speed of the beam is affected by the steering speed, observation distance, acceleration, and observation angle [13], which can not be directly equivalent to the platform moving speed. Zhao et al. [14] proposed the velocity in the distance model of the SAR using a virtual equivalent velocity over distance, but there are fewer descriptions of the velocity characterization of the SAR motion process for beam steering in industrial-limited environments. For satellite-based and airborne beam steering SAR, the range Doppler algorithm [15], chirp scaling algorithm [16,17,18,19], range migration correction algorithm [19,20,21], polar coordinate algorithm [22], sub-aperture algorithm [23], and backpropagation method [24,25] have been proposed. Chang et al. [26] proposed an advanced method to suppress the range ambiguity based on blind source separation for spaceborne SAR. Distinguished from the flexible motion scenarios of satellite-borne and airborne SAR platforms, BF radar installation size and motion platforms are strictly limited. Due to the special working system of fixed-platform FPBS-SAR, the above SAR imaging method cannot be applied to SAR processing for the BF burden surface. Currently, the radar measurement equipment of a fixed platform mostly adopts rotational scanning to obtain the echo of one-dimensional distance contour, and two-dimensional imaging is synthesized by non-coherent superposition [27]. The method uses matched filter processing only for the distance dimension and real aperture processing for the azimuth dimension, which is not conducive to azimuthal resolution enhancement. To optimize the constant false alarm rate detector (CFAR) for the homogeneous clutter environment, cell-averaged CFAR (CA-CFAR), maximum-order CFAR (GO-CFAR), minimum-order CFAR (SO-CFAR), sequential-order statistical CFAR (OS-CFAR), and Bayesian CFAR [28,29,30] were proposed. For the BF environment burden surface imaging scenarios, the clutter and the noise power have the characteristic of stochasticity, and there are fewer clutter suppression methods for such scenarios.

To summarize, the main problems of the burden surface imaging are as follows. First, real aperture imaging cannot eliminate the distance migration effect of the target due to the radar steering, resulting in the target showing a diffuse state, which seriously affects its imaging quality. Second, the industrial scene beam-steering radar SAR imaging mechanism and the time and frequency domain representation of the imaging mechanism urgently need to be solved. Third, due to the special environment inside BF, noncoherent noise greatly affects results of the burden surface imaging.

This paper aims to solve the theoretical problem of FPBS-SAR for industrial-limited scenarios and to propose a spatial–temporal domain imaging method, and the main contributions and innovations can be summarized as follows.

- The motion characteristics of fixed-platform beam-steering radar are studied, and the target range–azimuth coupled distance history model under the sub-aperture of fixed-platform beam-steering radar is established.
- The azimuthal Doppler variation characteristics of the fixed-platform beam-steering process are analyzed, its spatial–temporal representation is deduced, and the transform domain compensation method is proposed.
- The methods of FMCW sub-aperture FPBS-SAR imaging and the TGMOL-CFAR incoherent noise suppression are proposed, and the imaging method proposed is verified by simulation tests and industrial data.

The follow-up of this paper is organized as follows: Section 2 discusses the industrial beam steering radar signal model for fixed platforms, proposes a distance history model, and derives the equivalent speed, azimuthal tuning frequency, and azimuthal bandwidth analytical expression. Section 3 proposes a sub-aperture beam-steering SAR imaging method and noncoherent clutter and noise suppression method. Section 4 verifies the imaging theory and method through experimental simulation and field measurement data. Section 5 discusses the simulation and validation of field measurement data. Section 6 summarizes this paper.

## 2. Industrial Beam Steering Radar Signal Modeling and Spatial Variation Characterization

**Figure 1:**
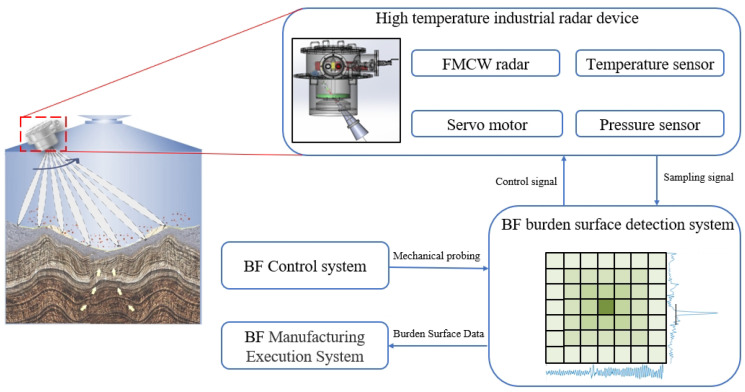
Beam- steering radar space detection.

In this paper, an industrial beam-steering FMCW-SAR system is used to drive the radar antenna movement by a servo motor to realize the antenna beam center steering control. Utilizing the antenna beam center steering obtains equal-angle uniform sampling of the BF limited space, realizing the original data acquisition. The beam-steering radar spatial detection process is shown in Figure 1.

In Figure 1, the blue area represents the upper space of the BF, the brown area represents stacking layer of ore and coke, and the light blue dashed line represents the beam scanning space.

### 2.1. Beam-Steering Radar SAR Imaging Mechanism Modeling

**Figure 2:**
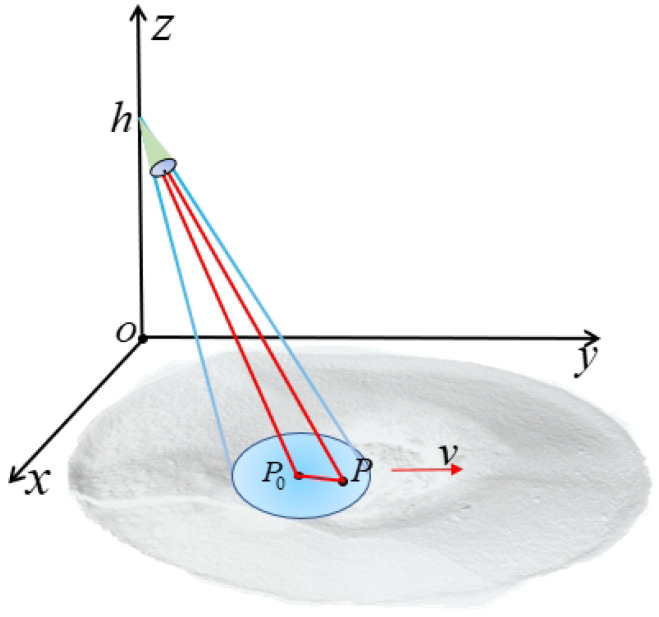
Imaging geometry of beam-steering radar.

The beam-steering radar geometric relationship is the fundamental of range history model. The Cartesian coordinate system is constructed according to the radar arrangement position; the target is located in P(x,y,z), the distance of the point target from the antenna when the beam center passes through the point target is $R(t_{r})$, the antenna length is L, the antenna rotation angular velocity is ω, and the radar scanning azimuth angle $\theta =\omega t_{a}$. The spatial geometric relationship of the beam-steering radar is shown in Figure 2.

In Figure 2, the green cone represents the radar antenna, the blue area represents the beam irradiation area, and the red line represents the geometric relationship between the target *P* and the beam center $P_{0}$.

For the BF FPBS-SAR radar, $R(t_{r})$ and the radar antenna length L no longer satisfy $R(t_{r})\gg L$, the relative position change between the radar and the target caused by the beam steering in the process of electromagnetic wave launching, receiving and propagating, so the instantaneous slant distance model between the radar and the point target P is described as $R(t_{r},t_{a})$.The inherent properties of nonlinear motion trajectories and imaging geometry relationships have a large impact on the accuracy of the slant range model. According to its geometric properties, $R(t_{r},\tau_{a})$ can be expressed as

(1) $$\begin{array}{c} R\left(t_{r},\tau_{a}\right)=\sqrt{R^{2}\left(t_{r}\right)+L^{2}-2LR\left(t_{r}\right)cos\left(\omega \tau_{a}\right)} \end{array}$$

$R(t_{r},t_{a})$ denotes the target distance course, $R(t_{r})$ is the reference slant distance when the beam center passes through the target, ω is the angular velocity of beam steering, $t_{r}$ is the distance time, and $\tau_{a}$ is the azimuth time.

### 2.2. Analysis of Accompanying Spatial Variation

The range history model is coupling with the range and azimuth so that it has two-dimensional coupling characteristics and does not have the characteristics of the explicit expression [17,31]. Because the analysis of the coupling characteristics and synthetic aperture processing has brought great inconvenience, this paper adopts the Taylor series approximation to the beam center irradiation of the target moments, which is expressed as (2).

(2) $$\begin{array}{c} R\left(t_{r},\tau_{a}\right)\approx R\left(t_{r}\right)+\frac{\omega LR\left(t_{r}\right)\sin\left(\omega \tau_{a}\right)}{R(t_{r},\tau_{a})}\tau_{a}+o\left(R\left(t_{r}\right)\right) \end{array}$$

**Figure 3:**
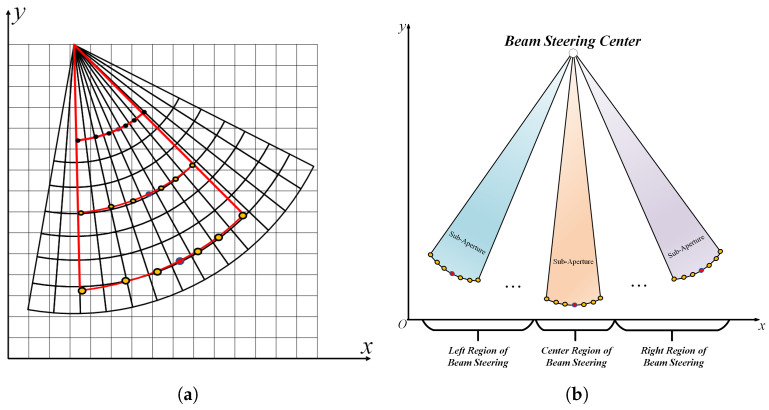
Range migration characteristics of point targets. (**a**) Sub-aperture range migration characteristics of point targets. (**b**) Sensing domain point target distance migration characteristics.

$o(R\left(t_{r}\right))$ denotes the target slant distance higher-order infinitesimal term. The migratory characteristics of the point target with range and azimuth are shown in Figure 3. The target migration increases with the radar steering away from the center of the synthetic aperture, and the range migration characteristic curve shows a quadratic-like distribution.The red line area in Figure 3a represents the synthetic aperture area.

### 2.3. Azimuthal Doppler Characteristics and Azimuthal Resolution

The beam-steering process causes a change in relative position, and the relative position causes a change in the distance history, so the beam steering regime radar cannot be interpreted as having no physical velocity of the target and the radar platform. To more accurately describe the physical significance of the distance course expansion terms of the beam steering process, the radar equivalent velocity $v_{eff}$ is proposed. For beam-steering SAR, the equivalent velocity mainly affects the synthesized aperture time, azimuthal Doppler frequency, and range migration term, which directly affect the beam-steering SAR image quality. The equivalent velocity is shown in (3).

(3) $$\begin{array}{c} v_{eff}=\frac{\omega LR\left(t_{r}\right)\sin\left(\omega \tau_{a}\right)}{R(t_{r},\tau_{a})} \end{array}$$

From (3), it can be concluded that the beam-steering SAR equivalent velocity is related to the beam-steering angular velocity, antenna length, and reference target range. Performing the Taylor approximation about the azimuth time $\tau_{a}$ in (3), the equivalent velocity and azimuth time change is a process of variable acceleration motion, and the approximate expression is shown in (4).

(4) $$\begin{array}{c} v_{eff}\approx \omega^{2}L\tau_{a}-\frac{1}{3}\omega^{2}L\tau_{a}^{3} \end{array}$$

FPBS-SAR azimuthal Doppler characteristics are different from strip, spotlight, and TOPS, and its azimuthal Doppler frequency is shown in (5). The FPBS-SAR azimuthal Doppler frequency is strongly related to beam-steering angular velocity and the antenna length.

(5) $$\begin{array}{c} f_{d}=\frac{2\omega LR\left(t_{r}\right)\sin\left(\omega \tau_{a}\right)}{\lambda R(t_{r},\tau_{a})} \end{array}$$

FPBS-SAR Doppler azimuthal tuning frequency is shown in (6).

(6) $$\gamma_{m}=-\frac{2\omega^{2}LR\left(t_{r}\right)R\left(t_{r},\tau_{a}\right)\cos\left(\omega \tau_{a}\right)-\omega LR\left(t_{r}\right)\sin\left(\omega \tau_{a}\right)v_{eff}}{\lambda R^{2}\left(t_{r},\tau_{a}\right)}$$

Azimuth resolution is an important parameter for near-field industrial SAR. The azimuthal Doppler bandwidth also has its unique representation due to the difference between the Doppler frequency and the modulation frequency and the conventional SAR radar due to the special way of FPBS-SAR operation, as shown in (7).

(7) $$B_{a}=\frac{2\omega^{2}LR\left(t_{r}\right)R\left(t_{r},\tau_{a}\right)\cos\left(\omega \tau_{a}\right)-\omega LR\left(t_{r}\right)\sin\left(\omega \tau_{a}\right)v_{eff}T_{d}}{\lambda R^{2}\left(t_{r},\tau_{a}\right)}$$

The azimuthal resolution reflects the minimum resolvability of the azimuthal dimension during beam steering, and FPBS-SAR azimuthal resolution expression (8) can be derived from (3) and (7).

(8) $$\rho_{a}=\frac{\lambda R^{2}\left(t_{r},\tau_{a}\right)}{2\omega^{2}LR\left(t_{r},\tau_{a}\right)\cos\left(\omega \tau_{a}\right)-\omega LR\left(t_{r}\right)\sin\left(\omega \tau_{a}\right)T_{d}}$$

## 3. FPBS-SAR Imaging Methods

FPBS-SAR analyzes the time–frequency domain of the transmit signal model and the echo signal model. First, FPBS-SAR-matched filtering and residual phase compensation functions are proposed. Second, the range–azimuth two-dimensional decoupling is realized by KT, and the two-dimensional frequency-domain compensation function is proposed to achieve the azimuthal focusing. Finally, a sub-aperture-based FMCW FPBS-SAR frequency-domain processing procedure and imaging algorithm is proposed.

### 3.1. Radar Echo Model

FPBS-SAR adopts THE FMCW signal; A is the radar system gain, $T_{p}$ is the linear FM repetition period, $T_{pd}$ is the frequency-modulated duration, $K_{r}$ is the linear modulation frequency, and the radar transmits the signal as in (9).

(9) $$\begin{array}{c} S_{t}\left(t\right)=Arect\left(\frac{t}{T_{pd}}\right)\exp\left[j2\pi f_{c}t+j\pi k_{r}t^{2}\right],\;t\in \left[0,T_{p}\right] \end{array}$$

The transmitted signal is scattered from the target to the radar, and the return delay IS Δt. In this paper, we mainly consider the FPBS-SAR aperture synthesis processing, ignoring the electromagnetic scattering process loss, and the return is shown in (10).

(10) $$\begin{array}{c} S_{r}\left(t\right)=Arect\left(\frac{t}{T_{pd}}\right)\exp\left[j2\pi f_{c}\left(t+\Delta t\right)+j\pi k_{r}{\left(t+\Delta t\right)}^{2}\right] \end{array}$$

The system mixes the received signal directly with the transmitted signal to obtain the intermediate frequency signal $S_{IF}\left(t\right)$ in (11).

(11) $$\begin{array}{c} S_{IF}\left(t\right)=A\exp\left(-j2\pi f_{c}\Delta t\right)\exp\left(j\pi k_{r}t\Delta t\right)\exp\left(j\pi k_{r}\Delta t^{2}\right) \end{array}$$

The first exponent term represents the distance phase, the second exponent term represents the azimuthal Doppler phase, and the third exponent term represents the residual video phase (RVP).

### 3.2. Analysis of Echo Signals

Substituting the range Equation (11) yields the two-dimensional function $S_{IF}\left(t,\tau \right)$ of the signal after mixing of the FMCW FPBS-SAR echo signal, as shown in (12).

(12) $$\begin{array}{c} S_{IF}\left(t,\tau \right)=Arect\left(t\right)w_{a}\left(\tau \right)\exp\left(j\varphi_{1}\left(t,\tau \right)\right) \end{array}$$

$t_{r}$ denotes the echo time delay at the target reference distance, and $\varphi (t_{r},\tau )$ is the phase function of the echo signal, expressed as (13).

(13) $$\varphi_{1}\left(t_{r},\tau \right)=2\pi f_{c}\frac{2R(t_{r},\tau )}{c}-2\pi k_{r}t\frac{2R(t_{r},\tau )}{c}-\pi k_{r}{\left(\frac{2R(t_{r},\tau )}{c}\right)}^{2}$$

The FFT transform of (11) is performed to obtain the distance to the frequency domain, and the orientation to the time domain is shown in (14).

(14) $$\begin{array}{c} S_{IF}\left(f,\tau \right)=w_{a}\left(\tau_{a}\right)\sin c\left[\pi T_{r}\left(f-k_{r}\frac{2R(t_{r})}{c}\right)\right]\exp\left(-j\varphi_{2}\left(f,\tau \right)\right) \end{array}$$

$\varphi_{2}\left(f,\tau \right)$ in (14) is expressed as

(15) $$\begin{array}{c} \varphi_{2}\left(f,\tau \right)=4\pi \left(f+f_{c}\right)\frac{R\left(t_{r}\right)-\omega^{2}L\tau }{c}+4\pi k_{r}{\left(\frac{R\left(t_{r}\right)-\omega^{2}L\tau }{c}\right)}^{2} \end{array}$$

The range–azimuth matched filter and range residual phase (MF-RVP) compensation function $H_{1}$ are constructed, as shown in (16). MF-RVP can process the distance term, which can effectively improve the signal-to-noise ratio (SNR) of the range dimension, correct the signal distortion effect in the beam steering radar system, and make the term of range obtain a good focusing effect.

(16) $$\begin{array}{c} H_{1}=\exp\left(j4\pi \left(f+f_{c}\right)\frac{R(t_{r})}{c}\right)\exp\left(j4\pi k_{r}\frac{R^{2}\left(t_{r}\right)}{c^{2}}\right) \end{array}$$

After MF-RVP compensation, (17) is obtained.

(17) $$\begin{array}{cc} \; & \varphi_{3}\left(f,\tau \right)=-4\pi \left(f+f_{c}\right)\frac{\omega^{2}L\tau }{c}-8\pi k_{r}\frac{R\left(t_{r}\right)\omega^{2}L\tau }{c}+4\pi k_{r}{\left(\frac{\omega^{2}L\tau }{c}\right)}^{2} \end{array}$$

In (17), there is a coupling with the range and azimuth variable term, which greatly affects the azimuthal consistency focusing. To remove the coupling part, the range–azimuth coupling relation is eliminated by KT, and $\tau^{\prime }$ is the azimuthal time after KT to obtain the decoupled phase function (18).

(18) $$\begin{array}{c} \varphi_{4}\left(f,\tau^{\prime }\right)=-4\pi \frac{\omega^{2}L}{c}\left(f_{c}+2k_{r}R\left(t_{r}\right)\right)\tau^{\prime }+4\pi k_{r}{\left(\frac{\omega^{2}L\tau^{\prime }}{c}\right)}^{2} \end{array}$$

The decoupled phase function is decomposed into two terms, the first being the azimuthal linear migratory term with the coupling removed, and the second being the azimuthal bending term. According to the principle of stationary phase (POSP), one can obtain the azimuthal time $\tau^{\prime }$ and azimuthal frequency $f_{\tau }$ with Equation (19).

(19) $$\begin{array}{c} \tau^{\prime }=\frac{cR\left(t_{r}\right)f_{\tau }}{4k_{r}\omega^{4}L^{2}}+\frac{f_{c}}{2k_{r}\omega^{2}L}+\frac{R(t_{r})}{\omega^{4}L^{2}} \end{array}$$

Substituting (19) in (18), we obtain the radar echo signal two-dimensional frequency domain function phase expression (20).

(20) $$\begin{array}{c} \varphi_{4}\left(f,f_{\tau }\right)=A_{1}f_{\tau }^{2}+A_{2}f_{\tau }+A_{3} \end{array}$$

where

(21) $$\begin{array}{c} A_{1}=\frac{\pi c^{2}R^{4}\left(t_{r}\right)}{4k_{r}\omega^{6}L^{2}} \end{array}$$

(22) $$\begin{array}{c} A_{2}=\frac{2\pi R\left(t_{r}\right)f_{c}}{ck_{r}\omega^{4}L}-\frac{\pi R(t_{r})f}{\omega^{2}L}-\frac{2\pi R^{2}\left(t_{r}\right)}{\omega L} \end{array}$$

(23) $$\begin{array}{cc} \; & A_{3}=\frac{\pi f_{c}^{2}}{c^{2}k_{r}\omega^{2}}+\frac{4\pi k_{r}R^{2}\left(t_{r}\right)}{c^{2}\omega^{6}L^{2}}+\frac{4\pi f_{c}R\left(t_{r}\right)}{c^{2}\omega^{4}L}-\frac{2\pi f_{c}^{2}}{ck_{r}}-\frac{4\pi f_{c}R\left(t_{r}\right)}{c\omega^{2}L}-\frac{4\pi f_{c}R\left(t_{r}\right)}{c}-\frac{8\pi k_{r}R^{2}\left(t_{r}\right)}{c\omega^{2}L} \end{array}$$

In (20), $A_{1}$ is the coefficient of the azimuthal frequency-domain quadratic term, also known as the distance bending term. $A_{2}$ is the coefficient of the azimuthal frequency-domain primary term, also known as the range walking term. $A_{3}$ is the azimuth residual compensation term. According to the FPBS-SAR azimuthal frequency-domain characteristics, the frequency-domain compensation function is constructed as in (24) and (25).

(24) $$\begin{array}{c} H_{2}=\exp\left(-jA_{1}f_{\tau }^{2}\right) \end{array}$$

(25) $$\begin{array}{c} H_{3}=\exp\left(-jA_{2}f_{\tau }-jA_{3}\right) \end{array}$$

### 3.3. Methods of Sub-Aperture FPBS-SAR Imaging

FPBS-SAR, due to its special regime, convolves the beam with the spatial target during steering. The presence of nonlinear spatial sampling features in the Cartesian coordinate system leads to strong spatial nonconsistent distortion in the synthetic aperture processing, which are difficult to compensate consistently. However, the range–azimuth time-domain space presents the characteristics of spatially uniform sampling. Through range–azimuth time-domain sub-aperture processing, FPBS-SAR is divided into several overlapping subspaces, and the synthesized aperture time is shown in (26).

(26) $$\begin{array}{c} T_{s}=\frac{\lambda }{L\omega } \end{array}$$

**Figure 4:**
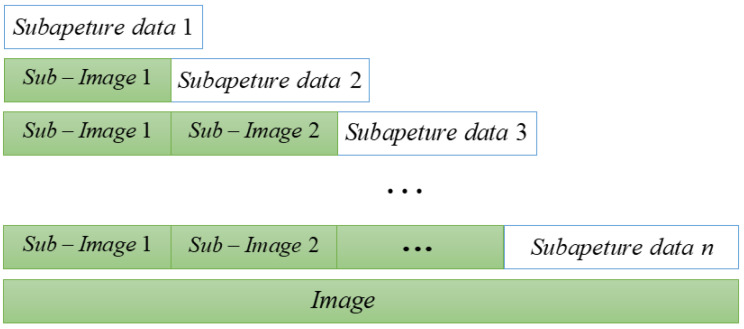
Divide of sub-aperture synthesis.

The sub-aperture division and sub-aperture synthesis are shown in Figure 4.

**Figure 5:**
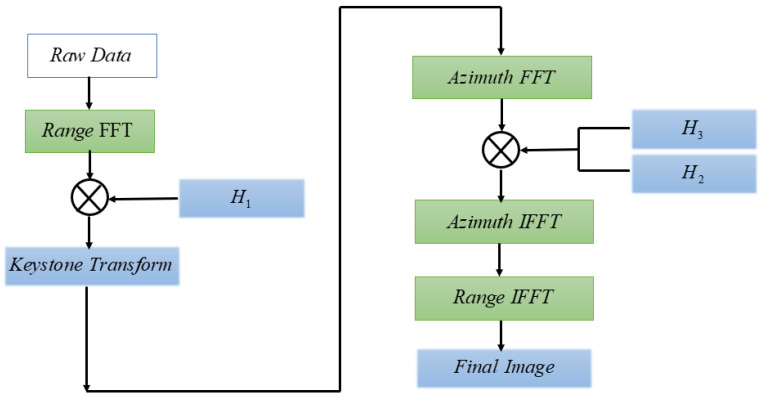
Block diagram of FPBS-SAR processing.

The flow of FPBS-SAR is shown in Figure 5. The system drives the FMCW radar to steer through the servo motor to realize equal-angle sampling and acquire the echo data. The overlapped sub-aperture data are divided into equal intervals, and the sub-aperture echo data are subjected to FFT processing, and the range dimension-matched filtering and residual phase compensation are realized by the MF-RVP compensation function $H_{1}$. After MF-RVP compensation from (17), there exists a range–azimuth coupling term. After KT, the decoupled radar echo data are obtained. Azimuthal dimension compensation under the sub-aperture is formed by two-dimensional frequency domain compensation functions $H_{2}$ and $H_{3}$, and the final sub-aperture imaging results are obtained after geometric correction.

### 3.4. TGMOL-CFAR

Nonconsistent sliding window detection processing, for the cell under tested (CUT) on both sides of the protection unit is set up on both sides of the protection unit, is clutter range profile (CRP)$x_{1},x_{2}\ldots x_{N}$. Adaptive thresholds are generated by CRP clutter evaluation. Target detection results are obtained by comparing the CUT with the adaptive threshold, eliminating the influence of clutter on imaging. For the BF burden detection scenario, the clutter environment and the clutter model scale function are unknown, which poses a great challenge for burden surface spatial clutter elimination. TGMOL-CFAR is proposed to realize clutter and noise suppression in the complex environment of BF by maximum likelihood estimation of noncoherent clutter parameters of FPBS-SAR echo data. The clutter environment in BF is assumed to satisfy a scale-unknown Weibull distribution. The probability density function is shown in (27).

(27) $$\begin{array}{c} f_{x}\left(x;b,c\right)=\frac{c}{b}{\left(\frac{x}{b}\right)}^{c-1}\exp\left[-{\left(\frac{x}{b}\right)}^{c}\right] \end{array}$$

For the case where the scale function of the clutter model is unknown, the core problem of CFAR is the problem of estimating the scale parameters with known shape parameters. TGMOL-CFAR is used to estimate the unknown scale factor, outliers are removed by ranking the CRPs, and the upper and lower CRP limits are determined by data preprocessing. The outlier judgment is shown in (28).

(28) $$\begin{array}{c} f\left(x_{i};{\hat{b}}_{t},c\right)>\frac{\alpha c}{N{\hat{b}}_{t}} \end{array}$$

α is the robustness factor, where the preprocessing part of the shape factor geometric mean scale factor estimate is as in (29).

(29) $$\begin{array}{c} {\hat{b}}_{t}={\left(\prod_{i=1}^{N}x_{i}^{c}\right)}^{\frac{1}{Nc}} \end{array}$$

After preprocessing the ordered-order CRP satisfies the equation ordered increment, the maximum-likelihood estimate of the scale factor for constructing the Wellbull distribution is shown in (30).

(30) $$\begin{array}{c} L\left(b|x_{r},c\right)=\sum_{i=N_{1}}^{N_{2}}a_{i}\ln f\left(x_{i};b,c\right) \end{array}$$

The maximum-likelihood estimate of the truncated geometric mean of the Weibull clutter shape parameter factorization is

(31) b^WL=(∑N1N2xic)1c

The false alarm probability density function is

(32) $$\begin{array}{c} P_{FA}=P\left(CUT>\tau f\left(X_{N_{1}},\ldots ,X_{N_{2}}\right)\right)={\left(1+\frac{\tau }{N}\right)}^{-N} \end{array}$$

where the detector multiplication factor is as in (33).

(33) $$\begin{array}{c} \tau =N(P_{FA}^{N}-1) \end{array}$$

The TGMOL-CFAR detector was derived from (31) and (33) as (34).

(34) $$\begin{array}{c} T\left(x\right)=\left(\frac{CUT}{{\hat{b}}_{WL}}\right)\underset{H_{2}}{\overset{H_{1}}{≷}}\;\tau \end{array}$$

**Figure 6:**
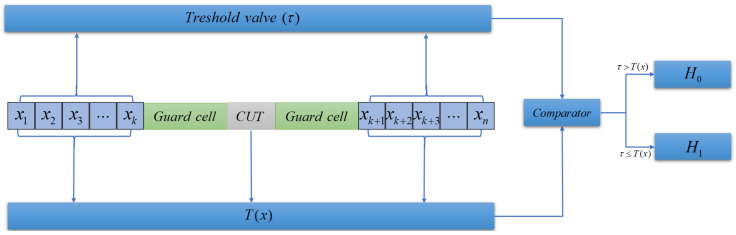
TGMOL-CFAR.

TGMOL-CFAR processing flow is shown in Figure 6, where it assumes that the CUT does not contain a target and indicates that the CUT contains a target.

## 4. Experiments

### 4.1. Simulation

In this part, the method proposed in this paper is validated by simulation. The beam-steering SAR radar simulation parameters are shown in Table 1, with a radar carrier frequency of 24 GHz, modulation bandwidth of 2 GHz, frequency modulation period of 25.5 ms, a beam steering angle of −30°–30°, the angular velocity of 2 rad/s, an antenna length of 0.3 m, and an antenna beam angle of 8°.

**Figure 7:**
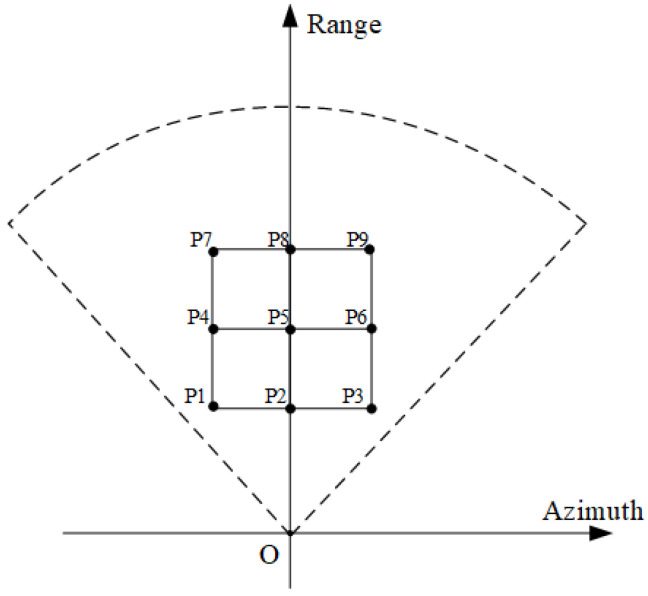
Point target distribution of simulation experiment.

The simulated point targets have a target distance of 10∼30 m and azimuth distance of −5 m∼5 m, and the point target arrangement is shown in Figure 7.

**Figure 8:**
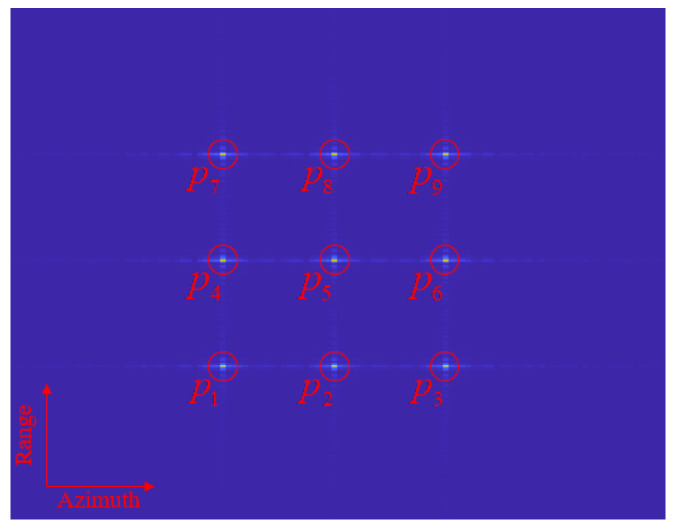
Simulation experiment point target imaging results.

**Figure 9:**
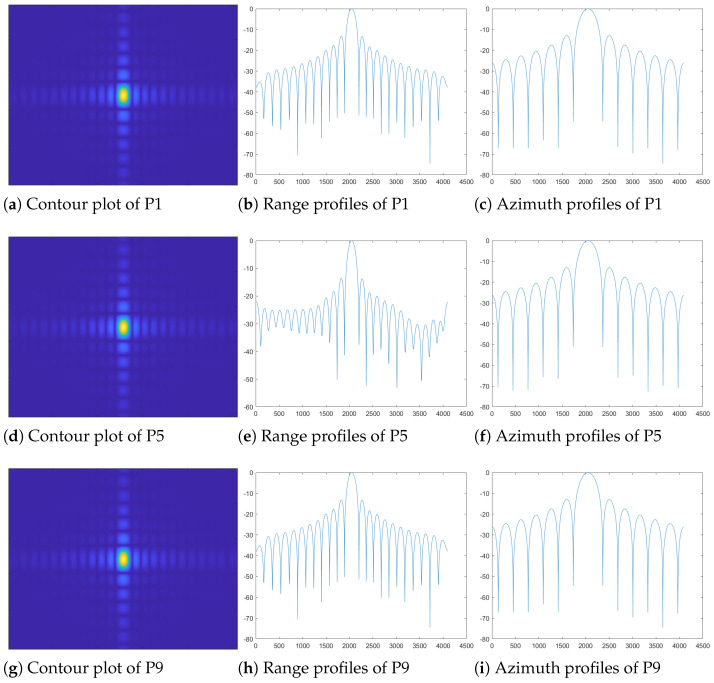
The imaging result of FPBS-SAR.

The point targets are uniformly distributed in the distance and azimuthal direction, and the simulation process ignores the shading before and after the point targets and the attenuation of the electromagnetic wave propagation in the space to better demonstrate the imaging effect in different range–azimuthal directions. The results of the FPBS-SAR imaging algorithm are shown in Figure 8. The simulated point targets have a target distance of 10∼30 m and azimuth distance of −5∼5 m, and the point target arrangement is shown in Figure 9.

By using the FPBS-SAR imaging algorithm, the imaging results for the chosen point targets are depicted in Figure 9. It should be highlighted that the SAR image in Figure 8 has undergone geometric correction processing, effectively eliminating geometric distortions.

The simulation performance evaluation of the proposed method in this paper is shown in Table 2, using peak sidelobe ratio (PSLR) and integral sidelobe ratio (ISLR) as evaluation metrics, respectively. The theoretical values of the PSLR and ISLR are −10.98 dB and −12.21 dB, respectively. Through the above experimental analysis, it is concluded that FPBS-SAR removes the two-dimensional coupling effect of the echo data by KT, the higher-order distorted terms are induced, and a good focusing effect is obtained.

**Figure 10:**
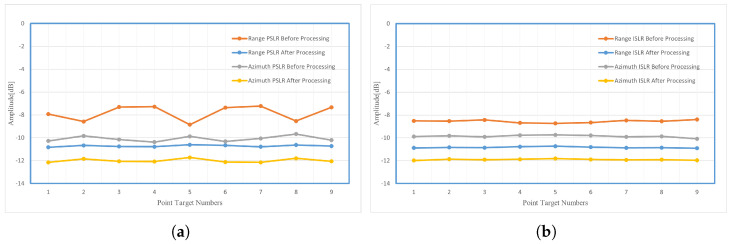
Comparison of range and azimuth dimensions indices of nine point targets before and after processing. (**a**) Comparison of PSLR indices of nine point targets before and after processing. (**b**) Comparison of ISLR indices of nine point targets before and after processing.

The comparison of range and azimuth dimensions indicators of the nine point targets before and after processing is shown in Figure 10. The PSLR and ISLR of point targets have been improved, indicating the effectiveness of the proposed method for point target scenarios. Especially for the process of beam steering that deviates from the center of the target, the degree of focus improvement is significant, which greatly alleviates the entropy increase of the target that deviates from the beam-steering center.

### 4.2. Industrial Data Validation

**Figure 11:**
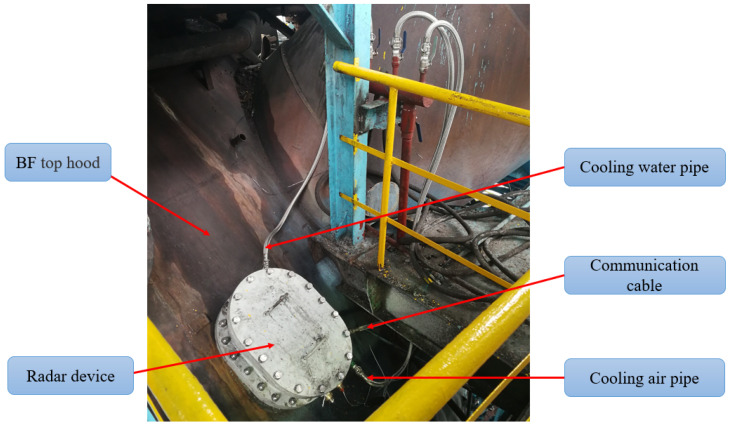
The site installation diagram of the radar.

To obtain the real-time burden surface, high-temperature resistance radar was installed on top of the BF top hood. Due to the high temperature and pressure inside BF, the radar device is equipped with water and air cooling to ensure that the working temperature of the radar device is below 60 °C. The air-cooled pipeline is simultaneously connected to the horn antenna to ensure that the pressure inside the radar horn is higher than the pressure of BF and to ensure that the radar antenna is not contaminated by strong dust. During the burden surface measurement process, the radar antenna steers to obtain the distance of the burden surface. The radar measures the distance between the burden surface and the radar at each scanning position correspondingly. The beam-steering radar installed atop the BF is depicted in Figure 11.

**Figure 12:**
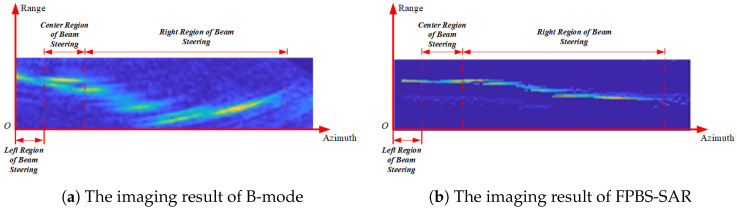
Comparison of BF burden surface imaging.

To further validate the effectiveness of the proposed method, which is validated by the measured data of the No. 7 BF burden surface of Wuhan Iron and Steel Company Limited, the B-mode and FPBS-SAR imaging results are shown in Figure 12.

The BF burden surface is a kind of random rough surface, and quality degradation seriously affects the burden surface target imaging results. This paper adopts image entropy (IE) and equivalent number of views (ENV) to evaluate the burden surface morphology characteristics; the performance indexes of B-mode and FPBS-SAR imaging results are shown in Table 3.

In Table 3, the IE and ENV of FPBS-SAR for the BF burden surface imaging results have been reduced, and the degree of focusing has been improved. The image distortion caused by beam steering is well corrected and the focusing performance of continuous shape targets on the burden surface has been improved. The field industrial data have verified the effectiveness of the proposed method for continuous surface imaging in enclosed limited spaces.

### 4.3. BF Clutter Suppression Validation

**Figure 13:**
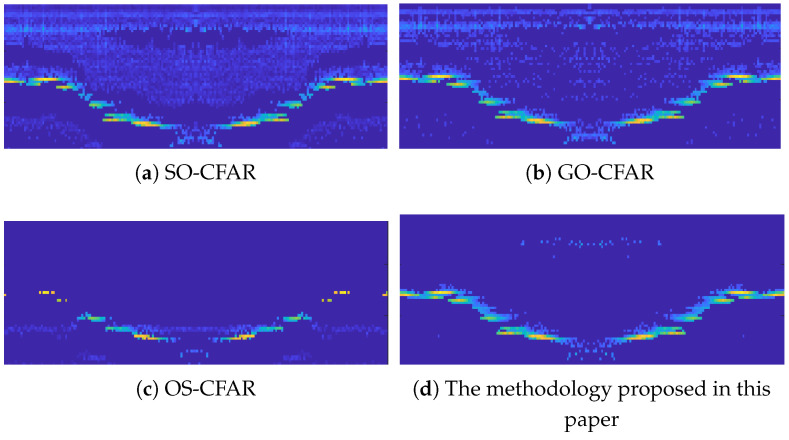
Effects of different CFAR.

For the problem of noncoherent noise suppression generated by the complex environment inside BF, the experiments are controlled against Smallest Of CFAR (SO-CFAR), Greatest Of CFAR (GO-CFAR), Order Statistics CFAR (OS-CFAR), and TGMOL-CFAR, and the results of the experiments are shown in Figure 13.

## 5. Discussion

For the scenarios of random rough surface measurement in the limited space, the installation space and location of the detection device are limited, and the detection is subject to many unexpected noises and clutter interference. The fixed-platform beam steering radar imaging results produce geometric distortion and are affected by incoherent noise.

The traditional beam-steering real aperture radar and B-mode imaging method have better imaging effects in the center area of beam steering. When deviating from the imaging center area, geometric distortion occurs. As the angle of deviation from the imaging center area increases, the geometric distortion becomes more severe, as shown in Figure 12a. Due to the lack of consideration of fixed-platform beam-steering radar aperture synthesis in real aperture radar and B-mode, the image entropy and equivalent number of views are high, and the image of the burden surface presents a certain degree diffusion status, resulting in distance blur, which affects the imaging effect and accuracy. In Figure 10a, it can be seen that the imaging results are worse for point targets 1, 3, 4, 6, 7, and 9 before FPBS-SAR processing, compared to point targets 2, 5, and 8, because of location in areas off the region of the beam-steering center. After the FPBS-SAR processing, the PSLR of point targets has been reduced, and the targets are well focused. In Figure 10b, the ISLR of the point target is reduced before and after processing, reflecting a decrease in the energy outside the main lobe resolution unit of the target after processing. In Figure 12a, deviating from the imaging center area, geometric distortion occurs, and as it moves away from the center region of beam steering, the geometric distortion further strengthens. After FPBS-SAR processing, the geometric deformation has been well corrected through the frequency-domain Keystone transformation in Figure 12b. For the comparison between the B-mode method and the FPBS-SAR method, as shown in Table 3, both IE and ENV were reduced, and the imaging diffusion effect and distance ambiguity of the burden surface were reduced.

For the suppression of incoherent clutter and noise, there is a certain degree of overlap between the boundary of the BF burden surface and the clutter. After processing with methods in Figure 13a,b, although the burden surface information is well preserved, the suppression effect is poor. For the method in Figure 13c, clutter and noise are well suppressed, while there is a significant loss of burden surface information. The processing effect of the method proposed in this paper is shown in Figure 13d, which achieves good suppression of clutter and noise while ensuring low loss of material surface information and achieving better imaging results.

In summary, the accurate and efficient FPBS-SAR algorithm provides superior imaging quality compared to the traditional BF radar imaging algorithm. The accuracy of the slant range model contributes to compress the history of beam steering, while the imaging method ensures efficient processing. This combination of BS-SAR and TGMOL-CFAR makes the algorithm well suited for imaging tasks and noncoherent noise suppression.

## 6. Conclusions

In this paper, the FPBS-SAR imaging method and TGMOL-CFAR incoherent clutter suppression method are proposed. The sub-aperture fixed-platform beam-steering radar range–azimuth coupled distance history model is established. It compensates for the IE and ENV increase and geometric deformation caused by the beam-steering radar of the fixed platform. Through the PSLR and ISLR of the simulation experiments, the proposed method in this paper obtains a better focusing effect for different range–azimuth point targets. Industrial data tests show that the IE is reduced from 6.8454 to 6.5835 and the ENV is reduced from 24.55 to 23.33 compared to the conventional method, the geometric aberration is well corrected, and the incoherent noise is well suppressed.

Considering the harsh environment and strong airflow interference in industrial limited spaces, there may be issues such as radar motion trajectory errors and data loss. In the future, further research will be conducted on the autofocus method for radar burden surface imaging under radar motion trajectory errors and the imaging method of burden surface under data loss, in order to improve the imaging ability of targets in harsh industrial environments.

## Figures and Tables

**Table 1:** Main parameters of the radar system.

Parameters	Valve
Carrier frequency/GHz	24
Range bandwidth/GHz	2
Frequency modulation rate/MHz/μs	80
Modulation duration/μs	25
ADC sampling frequency/MHz	25
Pulse repetition frequency/Hz	40
Pulse width/ms	25.5
Antenna beam angle/deg	8
Antenna length/m	0.3
Antenna steer angle/deg	−30°–3°
Antenna steer speed/rad/s	0 2

**Table 2:** Simulated imaging metrics of the nine point targets by the proposed method.

Target	PSLR (dB)		ISLR (dB)	
	Range	Azimuth	Range	Azimuth
P1(10,5)	−10.68	−11.95	−10.87	−11.87
P2(10,10)	−10.83	−12.15	−10.90	−11.98
P3(10,15)	−10.70	−12.06	−10.86	−11.88
P4(20,5)	−10.73	−12.08	−10.77	−11.91
P5(20,10)	−10.80	−12.18	−10.88	−11.81
P6(20,15)	−10.66	−12.02	−10.81	−11.93
P7(20,5)	−10.74	−12.08	−10.87	−11.89
P8(20,10)	−10.79	−12.15	−10.91	−11.96
P9(20,15)	−10.72	−12.06	−10.84	−11.91

**Table 3:** Comparison of B-mode and FPBS-SAR imaging results.

Model	IE	ENV
B Mode [10]	6.8454	24.55
Arc-SAR [28]	6.7235	24.02
FPBS-SAR	6.5835	23.33

## Data Availability

The data supporting the conclusions of this article are available from the authors upon reasonable request. The data are not publicly available due to privacy reasons.
